# Endozoochorous dispersal of forest seeds by carnivorous mammals in Sierra Fría, Aguascalientes, Mexico

**DOI:** 10.1002/ece3.6113

**Published:** 2020-02-19

**Authors:** Fabián A. Rubalcava‐Castillo, Joaquín Sosa‐Ramírez, José J. Luna‐Ruíz, Arturo G. Valdivia‐Flores, Vicente Díaz‐Núñez, Luis I. Íñiguez‐Dávalos

**Affiliations:** ^1^ Centro de Ciencias Agropecuarias Universidad Autónoma de Aguascalientes Aguascalientes México; ^2^ Departamento de Ecología y Recursos Naturales Centro Universitario de la Costa Sur Universidad de Guadalajara Autlán de Navarro Jalisco México

**Keywords:** *Canis latrans*, endozoochory, scats, seed dispersal, *Urocyon cinereoargenteus*

## Abstract

Some carnivorous mammals ingest fruit and disperse seeds of forest plant species capable of colonizing disturbed areas in ecosystems. The objective of the present study was to evaluate the dissemination of *Arctostaphylos pungens* and *Juniperus deppeana* seeds by the gray fox (*Urocyon cinereoargenteus*), coyote (*Canis latrans*), and other carnivores in the Protected Natural Area Sierra Fría, in Aguascalientes, Mexico. Scat collection was undertaken via transects using the direct search method, while the seasonal phenology of *A. pungens* and *J. deppeana* was evaluated by recording flower and fruit abundance on both the plant and the surrounding forest floor ground. Seed viability was assessed by optical densitometry via X‐ray and a germination test. It was found that the gray fox, coyote, ringtail (*Bassariscus astutus*), and bobcat (*Lynx rufus*) disseminated seeds of *A. pungens* (212 ± 48.9 seeds/scat) and *J. deppeana* (23.6 ± 4.9 seeds/scat), since a large proportion of the collected scat of these species contained seeds (28/30 = 93.33%, 12/43 = 27.9%, 6/12 = 50% and 7/25 = 28% respectively). The gray fox, coyote, ringtail, and bobcat presented an average of seed dispersion of both plant species of 185.4 ± 228.7, 4.0 ± 20.0, 12.1 ± 30.4, and 0.8 ± 1.5 per scat; the seed proportions in the gray fox, coyote, ringtail, and bobcat were 89.6/10.4%, 82.3/17.7%, 90.4/9.6%, and 38.1/61.9% for *A. pungens* and *J. deppeana*, respectively. The phenology indicated a finding related to the greater abundance of ripe fruit in autumn and winter (*p* < .01). This coincided with the greater abundance of seeds found in scats during these seasons. Endozoochory and diploendozoochory enhanced the viability and germination of the seeds (*p* > .05), except in those of *A. pungens* dispersed by coyote. These results suggest that carnivores, particularly the gray fox, the coyote, and the bobcat, play an important role in forest seed dissemination, and thus forest regeneration, by making both a quantitative and qualitative contribution to the dispersal of the two pioneer species under study.

## Graphical abstract

In the Sierra Fría, south of the Sierra Madre Occidental of México the most important pioneer species in forest regeneration and those that have had a greater distribution are: *Arctostaphylos pungens* and *Juniperus deppeana*. Our results indicate that these plant species are dispersed by the grey fox (*Urocyon cinereoargenteus*), coyote (*Canis latrans*), cacomixtle (*Bassariscus astutus*) and the wildcat (*Lynx rufus*). Likewise, these mammals were enhanced the viability and germination of *J. deppeana*. The abundance of fruit in the canopy was related to the abundance of seeds present in the scat during the season of autumn.

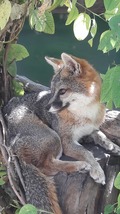

## 1 INTRODUCTION

In numerous plant species, seed dispersal is achieved via the process of endozoochory, in which plants produce fleshy nutritious fruits for consumption by animals, which then excrete the seeds at a distance from the parent plant (Cypher & Cypher, 1999). Various studies show that, in the ecological context of abandoned fields and degraded areas, frugivorous land mammals are principally responsible for seed dispersal and regeneration of vegetation (Escribano‐Ávila et al., 2014, 2012; Suárez‐Esteban, Delibes, & Fedriani, 2013). These studies reveal that scat distribution and deposition varies according to landscape, ecosystem, and disperser, while previous studies suggest that carnivores often defecate along paths and trails used by human beings (Fedriani, Palomares, & Delibes, 1999). Moreover, Suárez‐Esteban et al. (2013) suggest that paths and trails are corridors for plant species dispersed by mammals through their selection of these sites for defecation, which may act to promote native seed dispersal.

In this context, Zúñiga, Muñoz‐Pedreros, and Fierro (2008) describe how, although carnivore species are opportunists that mainly consume rodents and lagomorphs, they diversify their diet with birds, arthropods, fish, reptiles, and considerable quantities of fruit, the seeds of which are subsequently dispersed. For this reason, carnivorous species constitute an indispensable element of the dispersion guild of many plant species in highly anthropized environments (López‐Bao & González‐Varo, 2011; Perea, Delibes, Polko, Suárez‐Esteban, & Fedriani, 2013). Carnivorous mammals cover large areas and retain seeds for long periods in the intestine, making them key vectors for long‐distance dispersal (Jordano, Garcia, Godoy, & Garcia‐Castano, 2007; Otani, 2002). As a consequence, seed dispersal also benefits from the seed retention time and the transit time in the digestive tract (Cypher & Cypher, 1999), which may also have a beneficial effect on germination (Murray et al., 1994). Few studies explain the contribution of carnivores to the regeneration of vegetation; however, González‐Varo, López‐Bao, and Guitian (2013) demonstrate how the carnivores, red fox (*Vulpes vulpes*) and marten (*Martes martes*), play a crucial role in the long‐distance dispersion of seeds in temperate ecosystems. Likewise, Nakashima, Inoue, Inoue‐Murayama, Sukor, and J. (2010) show that the carnivorous mammal palm civet (*Paradoxurus hermaphroditus*) plays a unique and important role in the regeneration of *Leea aculeata*.

There is increasing interest in diplochory (two‐phase dispersion, also known as "secondary dispersion" or "indirect dispersion"), which involves a second phase of dispersal. However, relatively little attention has been given to "diploendozoochory," that is, seed dispersal involving ingestion of the seed by two or more different species of animals in sequence, generally a prey animal and its predator (Hämäläinen, 2017). Due to the complexity of the diploendozoochoric process, there is a shortage of worldwide studies of this mechanism. Of the few investigations in this area, one carried out by Sarasola, Zanón‐Martínez, Costán, and Ripple (2016) that involves a hypercarnivorous mammal recognized for its apex predator role: the puma (*Puma concolor*), which usually ingested and dispersed over long distances, large quantities of seeds of herbaceous species initially consumed by its main prey: Dove (*Zenaida auriculata*), with this, it was possible to prove that this feline plays the role of secondary seed disperser and demonstrate that strictly carnivorous predators such as felines could have extensive ecological functions.

The particular use of specific microhabitats and landscape elements by mammals, for example, for sleeping, reproduction, hunting, and shelter, determines seed deposition patterns (Russo, Portnoy, & Augspurger, 2006), which greatly affect the probability of subsequent recruitment of the dispersed seeds (Escribano‐Ávila et al., 2014). Escribano‐Avila et al. (2013) state that adaptation of a microhabitat for the germination and early survival of an animal‐dispersed seed depends on its selection, handling, and the effect of passage through the animal gut, in addition to the suitability of climatic conditions for triggering germination in the microsite, including temperature, humidity, light, oxygen, and even the surface type (Guariguata, 1999).

In Aguascalientes, Mexico, the *Área Natural Protegida Sierra Fría* (the Sierra Fría Protected Natural Area or ANP‐SF by its Spanish acronym) includes a group of ecosystems belonging to three biogeographic provinces (Sosa Ramírez, Breceda‐Solís, Jiménez‐Sierra, Iñiguez‐Dávalos, & Ortega‐Rubio, 2016). Díaz‐Núñez, Sosa‐Ramírez, and Pérez‐Salicrup (2016) describe the recovery of these ecosystems since the 1990s, reporting that the pioneer species that present the greatest distribution and colonization of the area postdisturbance are the pointleaf manzanita or pingüica (*Arctostaphylos pungens* Kunth; Ericaceae), which is a fire‐adapted plant that thrives in places where events have frequently destroyed holm oak trees (Rzedowski, 1978), and the checkerbark juniper or táscate (*Juniperus deppeana* Steud; Cupressaceae), which is a dioecious species that lives in temperate to semiarid environments and is tolerant to alkaline and nutrient poor soils (Batis, Alcocer, Gual, Sánchez, & Vázquez‐Yánez., C., 1999; Martínez, 1963). Both of these plant species act as nurse species to other larger species. The objective of this study was to evaluate the dissemination of seeds of *A. pungens* and *J. deppeana* by the gray fox (*Urocyon cinereoargenteus*), coyote (*Canis latrans*), and other carnivores in the ANP‐SF in order to determine whether these carnivores aid the regeneration of habitats. Specifically, we wished to determine whether the endozoochorous seed dispersal system in these mammals is one of the causes of the wide distribution in these plant species through the relation of the offer (abundance) of fruits in canopy with the abundance of seeds in scats and the record of the type of surface where the scats are deposited. In addition, we wished to explain the role (dispersant, scarifier, and germination promoter) of all mammals under study in the dispersion of the seeds of *A. pungens* and *J. deppeana* and whether bobcats also act as diploendozoochory vectors.

## 2 MATERIAL AND METHODS

### 2.1 Study site

**Figure 1:**
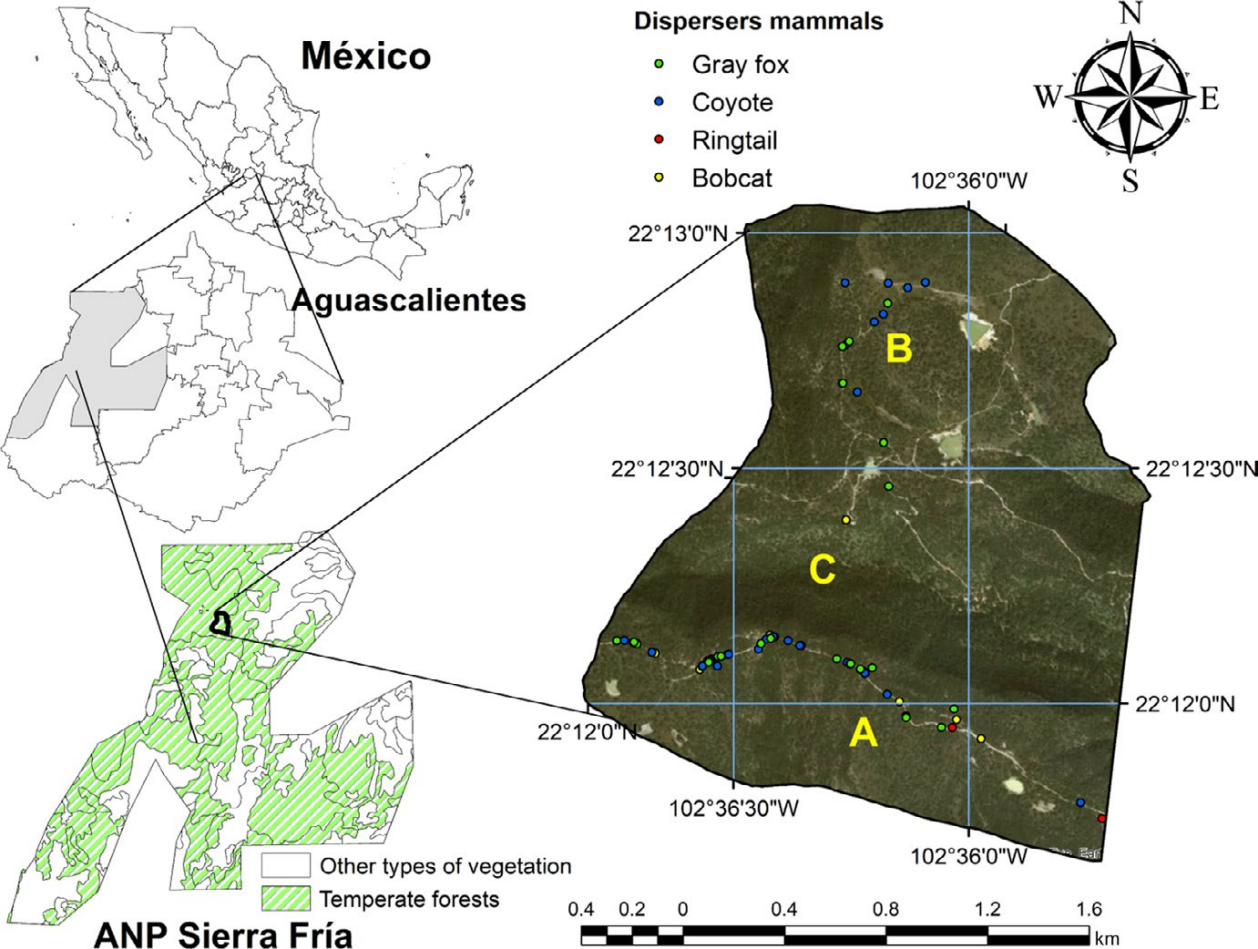
Geographical location of the study area in the temperate forest of the ANP‐SF in the state of Aguascalientes, Mexico. *Mesa del Aserradero* (a), *Mesa del Águila* (b), and *Cañada de Piletas* (c)

The study was carried out in an area of temperate forest in the ANP‐SF, on the western border of the State of Aguascalientes, Mexico. The area presents a subhumid temperate climate and summer rainfall (Rzedowski, 1978), with temperatures ranging from −3°C to 18°C and average annual rainfall fluctuating from 600 to 700 mm (SEDESO, 1995). The natural plant communities in the area are composed of *A. pungens*, *J. deppeana*, Oak (*Quercus potosina*), isolated elements of *Pinus leiophylla* var. *Chihuahuana* (a subspecies of the Chihuahua pine) and *Pinus teocote* (Díaz‐Núñez et al., 2012). Two areas were selected from within the ANP‐SF for collection of scats: *Mesa del Aserradero* and *Mesa del Águila*. *Mesa del Aserradero* is located at the coordinates 22°11′55.51′′N and 102°35′47.64′′W, while *Mesa del Águila* is found at coordinates 22°12′1.52′′N and 102°35′11.03′′W. These two plateaus are separated by the ravine *Cañada de Piletas*. Based on pre‐existing maps of the vegetation cover by Díaz‐Núñez et al. (2016), where, for the estimation of forest coverage, a SPOT^®^ 2013 satellite image of 5 meters resolution was used. The study area was divided into patches or fragments that form continuous mosaics within a landscape and four categories of coverage were established for the landscape classification: 1 = ≤10%, 2 = 11%–30%, 3 = 31%–50%, and 4 ≥50%. Image analysis was performed using ArcGIS^®^ 10.5 software (Environmental Systems Research Institute, 2011) of the ArcMap module, thus generating vegetation maps in which sites with coverage <40% were considered as open sites. Likewise, patches with coverage ≥40% were considered as closed sites. Based on the information on the previous maps and on direct field exploration of both plateaus, sites of closed vegetation (high cover) were identified, comprising numerous woody plants the canopies of which cover a large part of the forest floor, as well as open sites (low cover) with native grasses and very few woody plants (Figure 1).

### 2.2 Collection, identification, and location of scats

**Figure 2:**
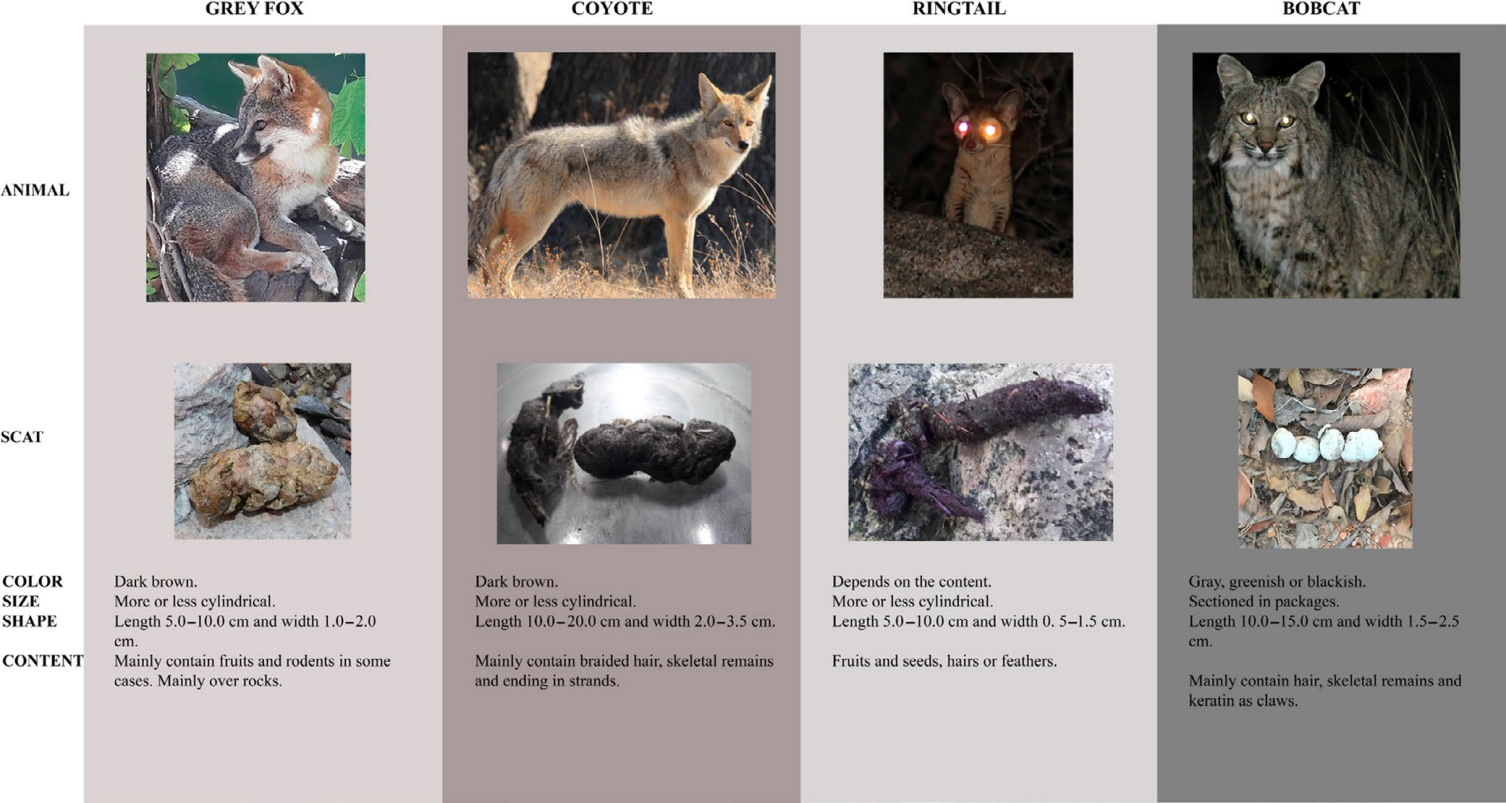
Description of representative scat with defined characteristics for the identification of each carnivore included in the analyses. The measurements and descriptions are based on Aranda‐Sánchez (2012)

Visits were undertaken at the study sites during all four seasons of both 2015 and 2016. Scat collection was carried out in each site and season via transects, using the direct search method proposed by Nova (2012), which consisted of making walking routes through the study area to locate scats at simple sight. Each transect consisted of a central line of 2 km in length, with two parallel lines located 20 m either side of the central line. The scat was then collected from the total area within the transect. The search for scats was performed in the entire study area dividing it by a grid of transects each separated by 20 m, trying to sample all types of surface. For the above, 72 transects were established on trails identified as routes for the movement of fauna, on dirt paths and among the vegetation in the area (both low and high cover), and the routes varied in order to cover as much of the plateaus as possible and to include all of the probable scat sites. Scat corresponding to each mammal species was identified based on the *Manual for Tracking the Wild Mammals of Mexico* (Aranda‐Sánchez, 2012) and immediately labeled. The main characteristics for identification were the shape and dimensions of the scat: gray fox scats are cylindrical in shape and are present on the rocks, forming latrines; coyote scats are formed mainly by hair in the form of braids and finished in a long lock; ringtail scats are thin and elongated, consisting of fruits, seeds, hairs, and feathers; and bobcat scats are usually in a cylindrical form and divided into several packages (Figure 2). Once identified, the scats were georeferenced using a GPS (Garmin, eTrex^®^10) and placed in crepe paper bags, with the type of surface on which it was found recorded using the abovementioned classification with the modification proposed by Matías, Zamora, Mendoza, and Hodar (2010), creating four distinct categories: (a) on‐unpaved road on rocks (unpaved road/rock); (b) on‐unpaved road on bare soil (unpaved road/bare soil); (c) off‐unpaved road on bare soil (off‐unpaved road/bare soil); and (d) off‐unpaved road on herbaceous vegetation (off‐unpaved road/herbaceous).

To identify the potential prey of the bobcat (*Lynx rufus*) and determine the incidence of diploendozoochory, the guard hairs contained in the scats of this feline were identified by consulting the guides for identification of mammal guard hairs by Pech‐Canche, Sosa‐Escalante, and Cruz (2009) and Monroy‐vilchis and Rubio Rodríguez (2014). For this, prior to identification, the hairs were subjected to a cleaning, clarification, and assembly process. The prey (species) was then identified with the use of an optical microscope (Leica Microsystems, DM LS2), considering patterns of hue, shape, maximum diameter, and hair marrow structure.

### 2.3 Identification and abundance of seeds

The scat was left to dry at ambient temperature (23°C) for 24 hr in Petri dishes, after which the seeds were extracted, washed under running water, and left to dry for 24 hr for subsequent analysis and identification. The seeds from each scat sample were quantified and then separated into three groups, according to plant species (*A. pungens*, *J. deppeana*, or other species). Identification took into account the distinctive characteristics present in *A. pungens*, the seeds of which are wedge‐shaped, an average of 3.2 mm in length and 2.6 mm in width and are sometimes united in groups of two to three (Márquez‐Linares, Jurado, & González‐Elizondo, 2006), and *J. deppeana*, the seeds of which are angular and irregular, 6 to 7 mm in length and 4 to 6 mm in width and light brown in color (Rzedowski & Rzedowski, 2005). This process was conducted using a stereoscope (Leica Microsystems, MZ6), obtaining the total number of seeds per scat sample and thus the richness of the species present, from which the abundance per plant species and per disperser animal species was determined.

### 2.4 Phenological analysis

Six 32 m × 32 m quadrants were installed in each vegetative cover and each plateau (three low and three high cover quadrants), that is, six in the *Mesa del Aserradero* and six in *Mesa del Águila*. Five *A. pungens* and five *J. deppeana* individuals of basal diameter >5 cm and height >2 m were selected and quantified per quadrant. The trees selected under these criteria corresponded to adult plants of reproductive age and thus had a high probability of bearing fruit. In total, 120 examples were counted (60 *A. pungens* and 60 *J. deppeana*) between the two plateaus. From autumn 2015 to summer 2016, according to the calendar for northern meteorological seasons, two visits per season were conducted per each included tree or sampled individual, at the beginning of, and halfway through, each season. On each occasion, the phenological variables described below were recorded, for each sampled individual.

Initial—recently pollinated flower, from which the corollas had fallen for *A. pungens*, with an incipient development of a small green fruit (immature stage), Unripe—green fruit fully developed (intermediate stage of development), and Ripe—fruit presenting red coloration typical of ripeness (reddish for *A. pungens* and brown for *J. deppeana*).

A modified version of the methodology proposed by Chapman et al. (1992) was applied to each of the sampled individuals in order to determine the abundance of flowers and fruit in the canopy. It is important to note that, for *J. deppeana*, only the abundance of fruits was determined since, as a gymnosperm, this species does not have flowers. Canopy volume was calculated for each sampled individual by measuring the longest axis from the crown and the axis perpendicular to this by extending a rope, marked at 1 m intervals, along the axis of the base of the tree. The height of the tree crown was measured using a clinometer. The canopy was then divided into four equal parts in the form of a Cartesian plane, on which imaginary lines were traced. Two 1 m^3^ areas were selected per quadrant (8 m^3^ in total), in which the number of flowers and fruit were counted separately for each estimated area. The average number of flowers and fruits for the two areas per quadrant was calculated; in this way, the general average for each of the four quadrants within an individual was calculated, in order to obtain a single general average of flowers and fruits for each sampled individual; finally, the general average per individual was used to calculate the total abundance of flowers and fruits based on the canopy volume previously calculated for each sampled individual.

The method described above was applied in order to determine the abundance of fruit on the forest floor, with the difference that the Cartesian plane generated here was applied on the forest floor surface below the canopy, calculating the average for the two areas, which this time comprised 1 m^2^ per quadrant, thus obtaining an overall average per sampled individual. In order to obtain the percentage abundance, the same calculation of the total abundance of flowers and fruits was applied based on the area on the ground below the canopy previously calculated, which was obtained by calculating the area of a circle created using the rope marked at one meter intervals to cover the entire canopy. All of the fallen fruit found on the ground pertaining to the sampled individual were included in the count.

### 2.5 Viability and germination test

A control group was established with seeds taken from mature fruit collected in the forest canopy of the study area for both the viability and germination tests. For this, 12 random individuals with ripe fruits were selected. For *A. pungens*, six specimens were chosen at *Mesa del Aserradero* and six at *Mesa del Águila*. For *J. deppeana*, 12 individuals with fruits were selected from a nearby area, since the production of this species was incipient in our study area. From each individual, ten ripe fruits were randomly collected and 30 seeds were subsequently selected. In this way, 360 seeds were obtained, representing the 12 individuals selected for each plant species. The viability test was conducted via optical densitometry analysis using X‐ray equipment (Faxitron X‐Ray Corporation, at 10 s and 22 kv intensity) using the technique proposed by De La Garza and Nepamuceno (1986). Viable seeds, with well‐developed embryos, were distinguished from nonviable seeds by the presence of mechanical damage and malformations, with the latter seed type either empty or without an embryo. The germination test was carried out in parafilm‐sealed Petri dishes (Antonio‐Bautista, 2012), placed in an incubation chamber (Lab‐Line, Model 310 Imperial III) at a temperature of 25°C for 63 days, with groups of 10 seeds per Petri dish, into which 6 ml of distilled water was poured prior to placement of the seeds.

### 2.6 Statistical analysis

The resulting data were captured in a database, based on which three statistical tests were performed using the Statgraphics (15.2, 2007) statistical program, with the values expressed as average ± *SD*. To analyze the seasonal dispersion by mammals, an analysis of variance (ANOVA) and Tukey's Honest Significant Difference (HSD) tests were then conducted to determine differences in the contribution of each mammal to the dispersal of seeds of both species, abundance of seeds per season of the year, and the total number of *A. pungens* and *J. deppeana* seeds dispersed separately. The *ab* averages with different literals in each variable presented statistically significant differences. Likewise, analysis of variance (ANOVA) and Tukey's Honest Significant Difference (HSD) tests were used to determine significant differences in the total seed dispersal for high and low vegetative cover, with a 95% significance level. In addition, the chi‐square test (*X* ^2^) of independence was conducted in order to test whether there is dependence on the dispersion carried out by mammals for dispersant plant species. To analyze the type of surface on which scat is deposited, the chi‐square test (*X* ^2^) of independence was also performed to verify whether there is dependence on the possible relationship between the deposition of scat (both on‐ and off‐unpaved roads) and the specific type of surface on which it is deposited (rocks, bare soil and herbaceous vegetation). Moreover, the Dunnett test was also conducted in order to determine significant differences in the viability and germination of seeds in the scat, compared with seeds collected from the canopy (control), to a 95% significance level. For the phenological analysis, analysis of variance (ANOVA) and Tukey's Honest Significant Difference (HSD) tests were also used to establish differences in the abundance of flowers and fruit in the canopy, as well as the fruit on the forest floor, per season and plant species, also at a 95% significance level.

## 3 RESULTS

### 3.1 Seasonal dispersal by mammals

Of the total mammalian scats found in the study area for a series of potential dispersers of, *A. pungens* and *J. deppeana*, only four mammal species pertaining to the carnivorous order and classified into three families (*Canidae*, *Procyonidae* and *Felidae*) were identified as potential dispersers by confirming the presence of seeds of these plant species in their scats. Seeds of *A. pungens* and *J. deppeana* were found in 110 scats of the total number of scats analyzed in four species of mammals: coyote (43 scat samples = 39.1%); gray fox (30 scat samples = 27.3%); bobcat (25 scat samples = 22.7%); and ringtail (*Bassariscus astutus* Lichtenstein) (12 scat samples = 10.9; Table 1). Of the scats of all four mammals, 22.7% contained seeds of *A. pungens* and 25.5% contained seeds of *J. deppeana*. It is important to note that, of the 25 scats found in wildcat, seven contained seeds of *A. pungens* (8 seeds) and *J. deppeana* (13 seeds). The seven scats with seeds all presented hairs and, through the use of the guide for identification of guard hairs, it was determined that the hairs of the prey belonged to the wild rabbit of the species *Sylvilagus floridanus.* Hairs of the genus of these rabbits have a multiserial medulla, a dark band and a shield shape.

**Table 1:** Data for each carnivore describing: number of scats analyzed, total average number ($\bar{x}$ ± *SD*) of seeds found, average number of seeds ($\bar{x}$ ± *SD*) found belonging to each plant species, number of germinated seeds, and total number of scats on each type of surface

Data	Dispersers				Total
	Gray fox	Coyote	Ringtail	Bobcat	
Scats analyzed (*N*)	30	43	12	25	110
Total No. of seeds dispersed	185.4 ± 228.7	4.0 ± 20.0	12.1 ± 30.4	0.8 ± 1.5	5,905
Dispersed seeds					
*A. pungens*	356.2 ± 238.5	20.5 ± 48.7	66.0 ± 56.5	4.0 ± 1.4	5,271
*J. deppeana*	41.1 ± 25.9	6.2 ± 6.3	3.5 ± 2.5	2.6 ± 1.6	634
Germination (*N*)					
*A. pungens*	0	0	0	0	0
*J. deppeana*	1	2	0	1	4
Surface on which scat is deposited (*N*)					
On‐unpaved road on rocks	17	23	5	17	62
On‐unpaved road on bare soil	10	14	6	5	35
Off‐unpaved road on bare soil	3	4	1	2	10
Off‐unpaved road on herbaceous vegetation	0	2	0	1	3

**Figure 3:**
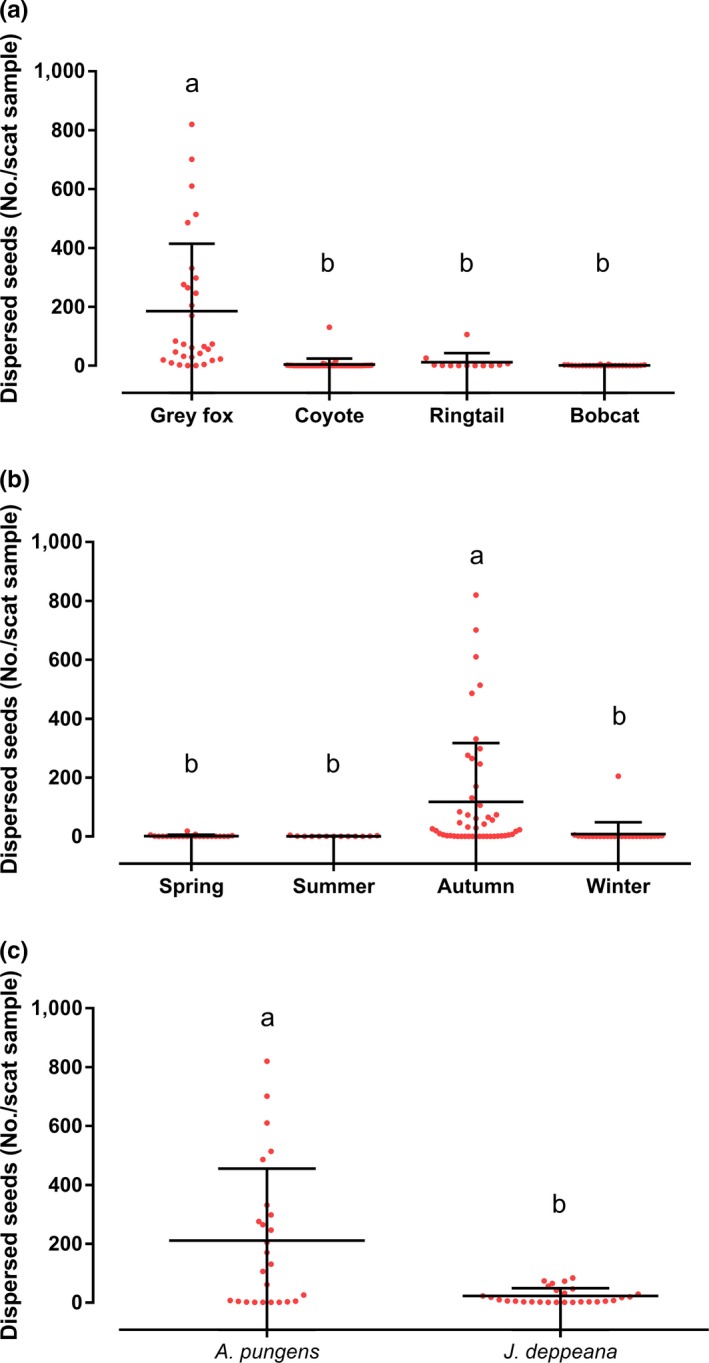
Dispersal of forest seeds in the ANP‐SF. Average dispersal (±*SD*) by the gray fox (*Urocyon cinereoargenteus*), coyote (*Canis latrans*), ringtail (*Bassariscus astutus*), and bobcat (*Lynx rufus*) (a). Average dispersal in each season (b). Average dispersal of the plant species *Arctostaphylos pungens* and *Juniperus deppeana* (c). ^a,b^Averages with different literals present statistically significant differences according to the Tukey HSD test (*p* < .01)

In terms of seed abundance, the four mammals dispersed a total of 5,905 seeds of both plant species in the study area. Specifically, the gray fox presented significant differences (*p* < .01, *F*_6, 109_ = 8.63, *p* = .00) by dispersing 94.2% of the total seeds of *A. pungens* and *J. deppeana* (5,563/5,905 = 94.2%), where it disseminated the highest average number ($\bar{x}$ ± *SD*) of forest seeds per total scat sample (185.4 ± 228.7), while the coyote dispersed 3% (4.1 ± 20), the ringtail 2.5% (12.2 ± 30.5), and the bobcat 0.3% (0.84 ± 1.6) (Figure 3a).

Six scat samples from other potential dispersers of these plant species were collected, including the mammals hooded skunk (*Mephitis macroura* Lichtenstein) (4/116 = 3.5%), raccoon (*Procyon lotor* Linnaeus) (1/116 = 0.86%), and weasel (*Mustela frenata* Lichtenstein) (1/116 = 0.86%). However, given that seeds of the plant species were not found in these scat samples, they were excluded from the analysis.

As in the case of disperser animals, a significant difference was found in general dispersal among the seasons of the year (*F*_3,112_ = 6.2, *p* = .00). This significant difference was found in the autumn season (111 ± 196), which presented 89.5% of the total seed dispersal (Figure 3b).

With regard to the abundance of *A. pungens* and *J. deppeana* seeds, separately, in the scat, a statistically significant difference was found for *A. pungens* (*F*_2,113_ = 32.1, *p* = .00). This was the most dispersed plant species in terms the total number of seeds per scat over the seasons, with 89.2% (211 ± 244.4), while *J. deppeana* recorded 10.8% (22.6 ± 26.2) (Figure 3c).

Statistically significant differences were observed in the chi‐squared independence tests (*p* < .05, *X^2^*_12_ = 42.9, *p* = .00) conducted among the four carnivore species and dispersed plant species, indicating that there is no independence in the dispersion of these seeds. In this way, there are different preferences of carnivores associated with the species of fruit they eat. The above is corroborated by the differences in the number of seeds dispersed by each mammal in the previous results.

Finally, the influence of the vegetative coverage on the total seed dispersal by the four mammals in closed (high cover) and open (low cover) sites was recorded, with no statistically significant differences presented by either cover (*F*_1,114_ = 3.39, *p* = .06), despite this, the highest average seed dispersal was recorded in the open sites (76.43 ± 190.8), compared with the closed sites (28.7 ± 68.1).

### 3.2 Phenological analysis

**Figure 4:**
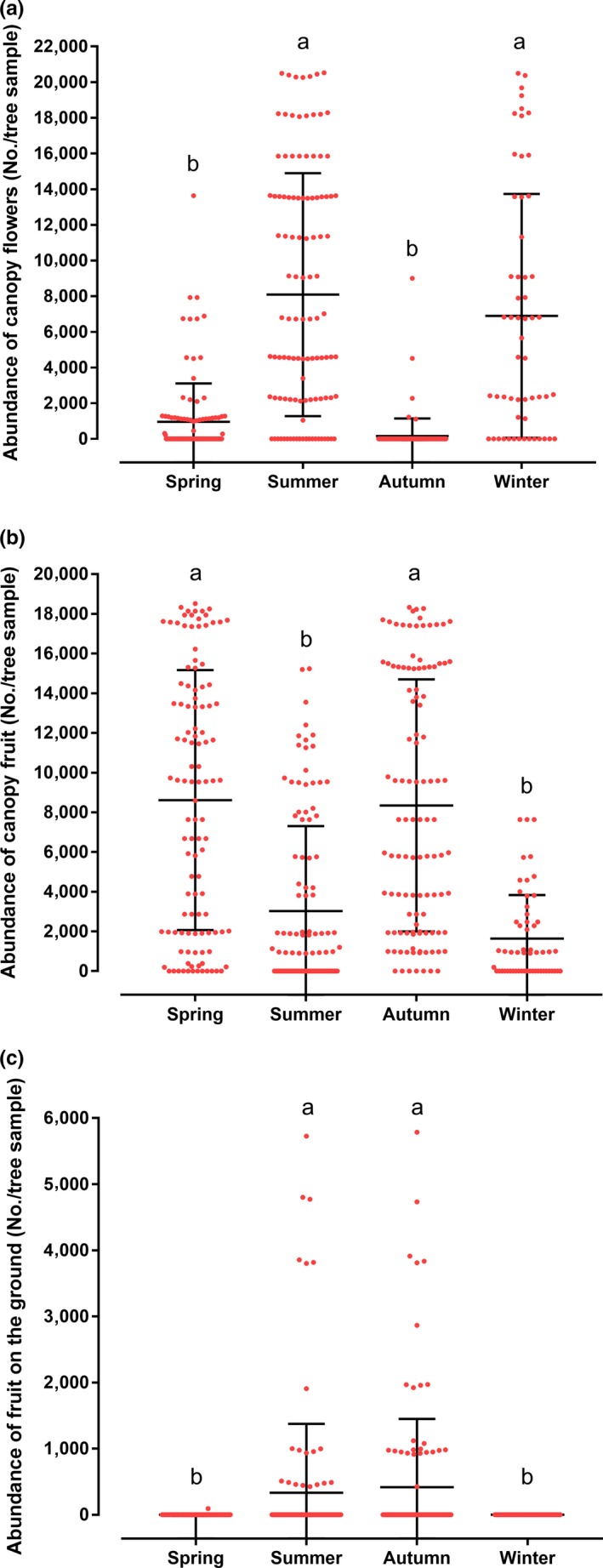
Phenological analysis of the *Arctostaphylos pungens* in the ANP‐SF. Average abundance (±*SD*) of flowers (a), average abundance of fruit in canopy (b), and average abundance of *A. pungens* fruit on the forest floor (c), during the four seasons of the year. ^a,b^Averages with different literals present statistically significant differences according to the Tukey HSD test (*p* < .05)

*Arctostaphylos pungens* presented two stages of flowering and fruiting during the study period. With regard to the abundance of flowers in the canopy, the seasons in which a higher average was presented (±*SD*), as well as statistically significant differences (*p* < .05, *F*_3,388_ = 77.0, *p* = .00), were summer (8,089 ± 6,808) and winter (6,895 ± 6,838) (Figure 4a). On completion, the two flowering periods were followed by two fruiting periods: spring (8,616 ± 6,547) and autumn (8,347 ± 6,348), and both seasons presented significant differences (*F*_3,388_ = 38.5, *p* = .00) and the highest average abundance of fruits (Figure 4b). Despite the presence of a large number of fruits in the canopies during the fruiting season, a low number of fruits was observed on the ground directly under the canopies, mainly in summer (333 ± 1,041) and autumn (419 ± 1,031), where significant differences were found in both seasons (*F*_3,388_ = 7.5, *p* = .00), (Figure 4c).

**Figure 5:**
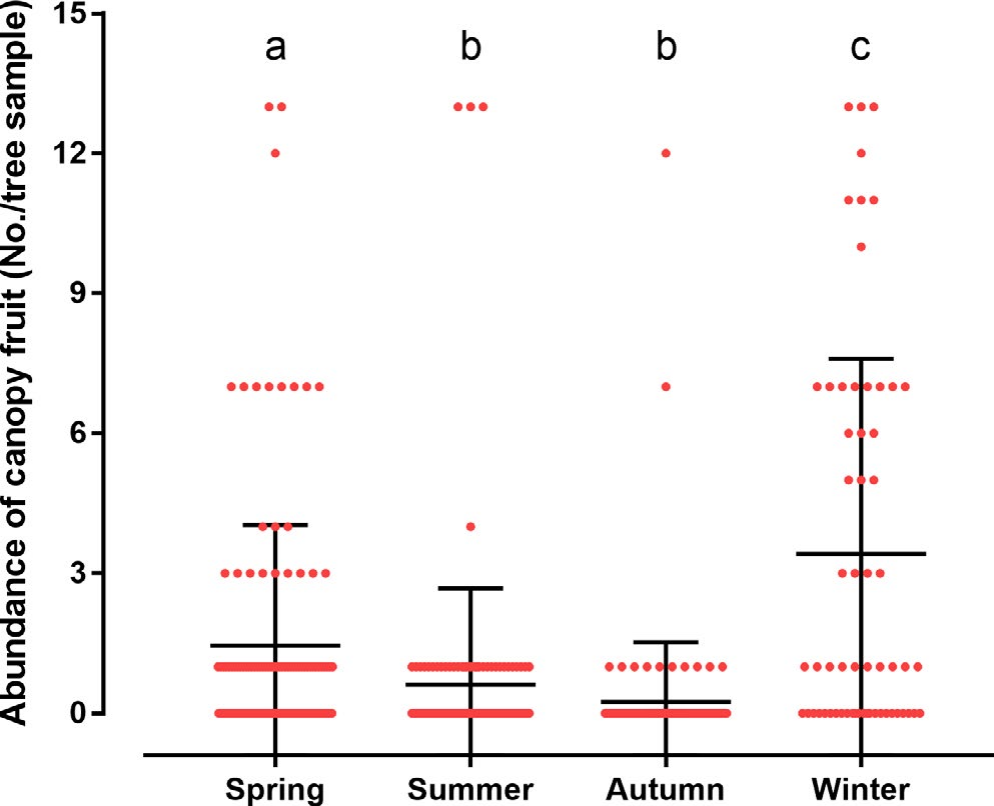
Phenological analysis of the *Juniperus deppeana* in the ANP‐SF. Average abundance (±*SD*) of fruit in the *J. deppeana* canopy during the four seasons of the year. ^a‐c^Averages with different literals present statistically significant differences according to the Tukey HSD test (*p* < .05)

Fruit production in the *J. deppeana* population was very low. However, the seasons in which there was a higher average abundance of fruits (±*SD*), as well as statistically significant differences (*F*_3,416_ = 24.83, *p* = .00), were spring (1.45 ± 2.57) and winter (3.41 ± 4.18) (Figure 5). No *J. deppeana* fruit were found on the forest floor. These results contrast with the abundance of *J. deppeana* fruit in the scat of the disperser species.

### 3.3 Type of surface on which scat is deposited

The spatial distribution of the scat showed that 87.9% was deposited on unpaved road used by motor vehicles, as can be seen in Figure 1. Of the scat samples collected on the unpaved road, 54.3% was found on rocks. Furthermore, statistically significant differences were revealed by the chi‐squared independence tests between the different types of surface on which the mammal scat was deposited and its placement on‐ or off‐unpaved roads (*X* ^2^_2_ = 41.0, *p* = .00), showing that there is dependence between the deposition of scat (both on‐ and off‐unpaved roads) and the specific type of surface on which it is deposited (rocks, bare soil and herbaceous vegetation); that is, the dispersers deposit their scats on a particular type of surface, in this case, on rocks on unpaved road (Table 1).

### 3.4 Viability and germination

**Figure 6:**
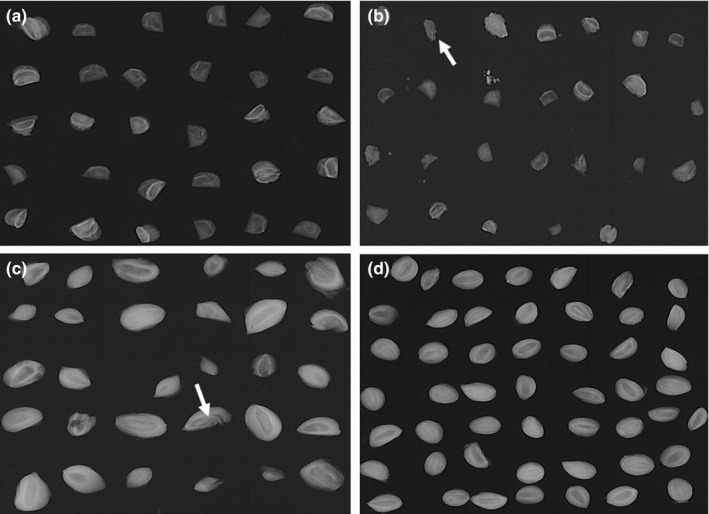
X‐ray test conducted on *Arctostaphylos pungens* and *Juniperus deppeana* seeds taken from both canopy and scats. *Arctostaphylos pungens* seeds taken from canopy (a) and from the scat (b), with the arrow indicating an incomplete seed with damage. *Juniperus deppeana* seeds taken from the canopy (c), with the arrow indicating the invasion of the screwworm in the embryo, and from the scat (d)

Endozoochory and diploendozoochory enhanced the viability of the *J. deppeana* seeds (*F*_4,29_ = 1.39, *p* = .26) since the passage of these seeds through the digestive tract of the gray fox (82.5 ± 13.6%), ringtail (84.1 ± 16.7%), and even bobcat (69.3 ± 41.3%) maintained the highest percentages of viability compared with seeds taken directly from the canopy (61.4 ± 12.3%). This is due to the fact that most of the canopy seeds presented perforations in their embryos caused by insects, lowering their viability compared with the seeds in the scats, which presented only minimal damage. The viability of *A. pungens* differed significantly between the seeds dispersed by the mammals and those taken from the canopy (*F*_4,17_ = 3.51, *p* = .02). This significance is reflected in the fact that the viability was only seen to be affected in the seeds obtained from coyote scat (45.2 ± 50.6%; Table 2). The X‐ray test showed that the *A. pungens* seeds taken from the canopy did not present damage or alterations (Figure 6a), while some of these seeds in the scat were either incomplete or presented some damage (Figure 6b). However, the seeds taken from the *J. deppeana* canopy presented a high level of damage caused by screwworms in the embryo (Figure 6c), while the *J. deppeana* seeds dispersed by mammals were found to be intact or with minimal damage (Figure 6d).

**Table 2:** Average number of viable seeds ($\bar{x}$ ± *SD*) for the viability test conducted via X‐ray in *A. pungens* and *J. deppeana* seeds taken from both the scat of the disperser mammals and the canopy

Seed species	Disperser species	Seeds (*N*)	$\bar{x}$ ± *SD*
*A. pungens*	Gray fox	54	21.5 ± 30.4
	Coyote	13	1.2 ± 1.9^a^
	Ringtail	26	18.0 ± 0.0
	Bobcat	5	3.0 ± 0.0
	Canopy (control)	360	29.2 ± 0.7
*J. deppeana*	Gray fox	257	24.1 ± 16.3
	Coyote	31	3.8 ± 3.4
	Ringtail	13	3.7 ± 2.1
	Bobcat	13	2.0 ± 1.6
	Canopy (control)	360	18.4 ± 3.7

a Statistically significant differences according to the Dunnett test (*p* < .05).

In *A. pungens*, no germination occurred in either the canopy seeds or those recovered from scats, despite waiting for the maximum period of 63 days. This may be due to the fact that this species has a very hard coat that prevented the entry of water necessary to trigger germination. Evaluation of the *J. deppeana* seeds (*F*_4,29_ = 0.89, *p* = .48) revealed that the mammals coyote (8.0 ± 17.9%) and bobcat (6.67 ± 14.9%) enhanced germination, presenting higher germination percentages than those of the seeds taken from the canopy (1.67 ± 4.1%). It can therefore be stated that ingestion acted to improve germination in this case (Table 1).

## 4 DISCUSSION

### 4.1 Quantitative contribution of carnivores (to the dispersal of pioneer species)

The gray fox was found to be the most important species in terms of the dispersal of seeds during all seasons of the year, due to the fact that its diet is almost entirely composed of fruit (Villalobos Escalante, Buenrostro, & Vega, 2014), including fruit of *A. pungens* and *J. deppeana*. The coyote was the species found to deposit the highest amount of scat, due to its wide distribution in the area. The ringtail, despite its inconsistent participation, also contributes to dispersal. However, while the diet of bobcat is considered essentially carnivorous (Sánchez‐González, Martin, Rosas‐Rosas, & García‐Chávez, 2018), the present study found a constant level of dispersal of the seeds of both plant species (*n* = 21). This species of feline is thus a secondary disperser, consuming, as prey, rabbit (*S. floridanus.*) that has consumed the fruit of *A. pungens* and *J. deppeana*. Thus, these seeds undergo a second endozoochorous process, ingested first by the rabbit and then by the bobcat, which is considered a diploendozoochorous disperser (Hämäläinen et al., 2017), as described by Sarasola et al. (2016) when seeds of herbaceous plants previously ingested by the Eared Dove (*Zenaida auriculata*) were found in puma scats. Likewise, Nogales, Medina, and Valido (1996) and Nogales, Castañeda, López‐Darias, Medina, and Bonnaud (2015) report the secondary dispersal of seeds by the feral cat (*Felis silvestris catus*), in which these authors describe seeds of plant species combined with the remains of lizard prey (*Gallotia galloti*) in the scats of this feline. These studies coincide with our study, in which seeds were found in scats of the bobcat, indicating that this feline is another diploendozoochorous mammal. However, further research is required in order to closely observe feeding in this and other potential felines in the region to confirm this diploendozoochory.

Some species of fauna, such as the coyote and ringtail, may occasionally act as disperser agents, when the high energy requirements of the animals oblige them to consume large quantities of plant resources if they are unable to secure their normal prey (Godínez‐Álvarez, Valiente‐Banuet, & Rojas‐Martínez, 2002). This implies that fruit consumption by the animal is strongly influenced by seasonality (Campos & Ojeda, 1997), and their dietary habits present a preference for the consumption of rodents or larger prey (Martínez‐Vázquez, María González‐Monroy, & Díaz‐Díaz, 2010; Rodríguez‐Estrella, Moreno, & Tam, 2000), although there is evidence of the consumption of seeds in their scat. For this reason, it is important to analyze the diet of species potentially involved in endozoochory in order to determine the quantity of seeds consumed. Such research would help to elucidate the trophic relationships that exist in the ecosystem, providing an approximation of the impact they could produce on the populations of plant species or the animals that consume them, in accordance with that established by Korschgen (1969).

The seasonality of the contribution of these mammals depends on prey and fruit availability. Therefore, with regard to the phenology of the plant species under study, we have proven statistically that the abundance of seeds in scat changes with the passing of the seasons, in which the highest numbers of dispersed *A. pungens* and *J. deppeana* seeds are presented in autumn. This abundance then decreases in winter due to the reduced presence of fruit in the canopy. It is likely that dispersal is related to the flowering and fruiting stages of the species analyzed, although it is not necessarily equal between these stages, as seen in the two annual fruiting cycles of *A. pungens* and the lack of *J. deppeana* fruit in the year of observation, as established by the phenological study. This suggests the existence of other sites in Sierra Fria with a different phenology for these plant species on which the fauna feed and subsequently transport into the study area (Luna‐Ruíz, Moreno‐Rico, Sosa‐Ramírez, & Sánchez‐Martínez, 2016). Similarly, our results contrast in seasonal terms, in that the phenology analysis strongly identifies two seasons (spring and autumn) that present an abundance of fruit, while the seeds contained in the collected scat are abundant in autumn only. This could indicate an abundance of other alternative prey in spring, for which reason the animals do not prioritize the consumption of fruit in this season. To verify the above, further research is recommended on parental assignment for dispersed seeds in scats in order to fully understand the seed flux.

### 4.2 Qualitative contribution of carnivores

It was observed that the viability of *J. deppeana* seeds remained intact during their passage through the digestive tract of the majority of the mammals studied, a finding similar to that of the study conducted by Aronne and Russo (1997), who conclude that *Myrtus communis* seeds dispersed by the red fox maintain their viability. Moreover, these authors also found that the low viability presented by the seeds taken from the canopy was attributable to the damage caused by parasitic insects, as reported by Martínez, Sainos, Lezama‐Delgado, and Angeles‐Álvarez (2007), who also observed damage by parasitic insects in *J. deppeana* seeds. Ingestion by the coyote particularly reduced the viability of *A. pungens* seeds, as reported by Matías et al. (2010), who determine a reduction in the viability of *Arctostaphylos uva‐ursi* seeds dispersed by the red fox. Nevertheless, the reduced viability of another plant species after ingestion by a different mammal species does not necessarily explain the results found for the coyote in this study.

The present study found that the disperser mammals enhanced germination of *J. deppeana*, particularly in seeds dispersed by coyote and bobcat, compared with that of the seeds taken from the canopy. This finding is similar to that of Graae, Pagh, and Bruun (2004), who reported increased germination in the species *Cerastium alpinum* and *Stellaria longipes* after passing through the digestive tract of the Arctic fox (*Alopex lagopus*). In *A. pungens*, germination did not occur, a result also found by Márquez‐Linares et al. (2006), who were unable to germinate this species from the scat of gray fox and coyote. However, these authors found that conditions mimicking those that occur during forest fires, such as certain temperature combinations (100°C and 120°C for five minutes), exposure to smoke and irrigation with water containing coal residues, can produce 29% germination.

The spatial distribution of scat over the landscape should also be noted, given that it has been shown that there are significant differences in the type of surface on which it is deposited, and that the majority was deposited on unpaved road with a preference for deposition on rocks. The coyote was the species that made the highest number of deposits in this microhabitat. In second place, scat was found on bare soil on unpaved road, while a small percentage was found off‐unpaved road, probably influenced by the ease of movement for dispersers in that microhabitat. Despite the fact that the majority was deposited on unpaved road, there is more opportunity for the germination of the seeds in the scat found on bare soil (both on‐ and off‐unpaved roads, with 33.6% and 8.6%, respectively) and on herbaceous vegetation (3.5%). We argue that 45.7% of the deposits via endozoochory are found in microsites suitable for the germination and establishment of plant species dispersed via this mechanism, provided there is no constant vehicle traffic over the unpaved road, which would eliminate the seeds that have germinated. Thus, the ideal scenario for germination would be if the seeds from the scat were deposited on bare earth (either on‐ or off‐unpaved road), than on rocks, given that seeds deposited on hard or easily‐dried surfaces face factors that inhibit germination and establishment, with some seeds recalcitrant, finding obstacles for establishment or even being transported to other sites via the wind (Guariguata, 1999). Thus, despite having sampled from all types of surface of the study area, we found that about 88% of scats were found on‐unpaved roads; however, it is clear that there is an establishment of these species of plants off‐unpaved roads, and therefore, it is necessary to carry out future research to understand if another dispersion system or factor is involved in carrying the seeds of scats that are on‐unpaved roads out of these for its germination and establishment.

Given the above, there is the possibility that a secondary dispersal system, via hydrochory, anemochory (Correa, Álvarez, & Stevenson, 2015), or even predation by mice (Escribano‐Ávila et al., 2014), causes the seeds found on‐unpaved roads to be transported beyond these paths to the surrounding areas of vegetation. In sites with bare soil (both on‐ and off‐unpaved roads), and even beyond the unpaved road in our study area, numerous seedlings are observed at the beginning of ecological succession, possibly as a result of these dispersal mechanisms. This could be associated with that reported by Suárez‐Esteban et al. (2013), who argue that dispersers selecting unpaved roads can promote roadside restoration. Thus, further research is recommended in order to analyze the secondary seed dispersal of seeds deposited on unpaved roads.

While the results are not presented, because the statistical analysis did not reveal significant differences in the influence of vegetation cover on scat distribution, we found that the highest number of seeds was dispersed in sites with low cover, namely open sites (< 49% canopy). These results concur with that described by Matías et al. (2010) in that mammals involved in endozoochory, such as the red fox, stone marten (*Martes foina*), and wild boar (*Sus scrofa*), disperse even more seeds in degraded habitats, generating a window of opportunity for seeds and seedlings via lower levels of competition and increased nutrient availability (Keeley, Keeley, & Bond, 1999). The relatively higher percentage of seeds dispersed in open sites found in our study therefore suggests that gray foxes, coyotes, ringtails and bobcats might also be dispersing seeds to degenerated habitats. Again, further research analyzing scats deposited within and beyond degenerated habitats of the area is required for confirmation.

The distribution and deposition of scat in the ANP‐SF varies according to the region and ecosystem, for which reason our results differ from that established by Escribano‐Ávila et al. (2014), who suggest that carnivores mainly deposit their scat under shrubs, thus generating a recruitment pattern for established Spanish junipers (*J. thurifera*). However, we do concur with this author in arguing that the excretion activity is the result of behavior related to the marking of territory. The present study found statistically significant differences in that the majority of deposits were made on the unpaved road, which is no coincidence, given that these appear to be sites favored by the mammals, as indicated by Suárez‐Esteban et al. (2013) when reporting that unpaved roads are favored by the arrival of seeds via the scats of mammals such as the red fox and the Eurasian badger (*Meles meles*). This conforms to the behavior of this group of animals involving the use of linear structures (pathways or unpaved road) as sites for displacement and marking of territory. It is worth mentioning that there is an underestimation of the number of scats and seeds found, due to the 20 m interval between each transect and the ease of finding scats on‐unpaved roads; however, although there is an underestimation, it does not represent a bias, so the numbers presented here are representative of what happens with mammals throughout the area.

These animals have the potential to disperse over long distances, and further research should determine the species‐specificity of seed dispersal distance (Gelmi‐Candusso et al., 2019). These behaviors correspond to a landscape that receives the seeds of many fleshy fruit shrubs (Suárez‐Esteban et al., 2013). It is probable that they do not always constitute a biological corridor factor; however, a high density of routes for the movement of fauna can act to increase or decrease the connectivity of habitats (Oliver & Larson, 1996).

## 5 CONCLUSIONS

Our results show that carnivores, particularly the gray fox, seem to contribute both quantitatively and qualitatively to the dispersal of the two studied pioneer species. However, the quantitative contribution of all carnivores is dependent on prey availability and fruit availability, which presents a seasonal pattern, and the qualitative contribution in terms of viability and germination seems to be highly species‐specific. Our results therefore support the hypothesis that carnivores are key for the dispersal of pioneer species, potentially even determining habitat regeneration. Future research should address seed dispersal distance by carnivore species in order to determine the spatial range of their influence.

## CONFLICT OF INTERESTS

None declared.

## AUTHOR CONTRIBUTIONS

The study was designed jointly by F.A.R.C. and J.S.R. F.A.R.C led this project and conducted all aspects of it, including collection of scats, laboratory work, and writing. J.S.R. acquired the funds and contributed significantly throughout the project with writing and corrections. J.J.L.R. collaborated in the methodological design, writing, and statistical analysis of viability and germination. A.G.V.F. contributed significantly to writing, monitoring graphics, and the statistical analysis. V.D.N. oversaw the reviews and analysis of the statistical data. L.I.I.D. collaborated in the revision, correction, and theoretical foundations.

## Data Availability

The sampling locations for each scat are available in Dryad (https://doi.org/10.5061/dryad.f7m0cfxrw).

## References

[ece36113-bib-0001] Antonio‐Bautista, A. (2012). Manual de Ensayos de Semillas Forestales Recopilación de Información. Coahuila. ISBN‐978‐607‐95357.

[ece36113-bib-0002] Aranda‐Sánchez, J. M. (2012). Manual para el Rastreo de Mamíferos Silvestres de México, Primera edn México, D.F.: CONABIO http://200.12.166.51/janium/Documentos/6800.pdf

[ece36113-bib-0003] Aronne, G. , & Russo, D. (1997). Carnivorous mammals as seed dispersers of *Myrtus communis* (Myrtaceae) in the Mediterranean shrublands. Plant Biosystems—An International Journal Dealing with All Aspects of Plant Biology, 131(3), 189–195. 10.1080/11263504.1997.10654181

[ece36113-bib-0004] Batis, A. , Alcocer, M. I. , Gual, M. , Sánchez, C. , & Vázquez‐Yánez, C. (1999). Árboles y Arbustos Nativos Potencialmente Valiosas para la Restauración Ecológica y la Reforestación. México, D.F.: Instituto de Ecología, UNAM‐Conabio.

[ece36113-bib-0005] Campos, C. , & Ojeda, R. (1997). Dispersal and germination of *Prosopis flexuosa* (Fabaceae) seeds by desert mammals in Argentina. Journal of Arid Environments, 35(4), 707–714. 10.1006/jare.1996.0196

[ece36113-bib-0006] Chapman, C. , Chapman, L. , Wangham, R. , Hunt, K. , Gebo, D. , & Gardner, L. (1992). Estimators of fruit abundance of tropical trees. Biotropica, 24(4), 527–531. 10.2307/2389015

[ece36113-bib-0007] Correa, D. , Álvarez, E. , & Stevenson, P. (2015). Plant dispersal systems in Neotropical forests: Availability of dispersal agents or availability of resources for constructing zoochorous fruits? Global Ecology and Biogeography, 24(2), 203–214. 10.1111/geb.12248

[ece36113-bib-0008] Cypher, B. , & Cypher, E. (1999). Germination rates of tree seeds ingested by coyotes and raccoons. The American Midland Naturalist, 71–76. 10.1674/00030031(1999)142[0071:GROTSI]2.0.CO;2

[ece36113-bib-0009] De la Garza, L. P. , & Nepamuceno, M. F. (1986). Análisis radiográfico de semillas forestales en México. Revista Ciencia Forestal, 11(59), 1–14.

[ece36113-bib-0010] de Rzedowski, G. C. , & Rzedowski, J. (2005). Flora fanerogámica del Valle de México, 2ª. edn, 1ª. Reimp. (pp. 1406). Pázcuaro, Michoacan: Instituto de Ecología, A.C. y Comisión Nacional para el Conocimiento y Uso de la Biodiversidad.

[ece36113-bib-0011] Diaz‐Núñez, V. , Sosa‐Ramírez, J. , & Pérez‐Salicrup, D. R. (2012). Distribución y abundancia de las especies arbóreas y arbustivas en la Sierra Fría, Aguascalientes, México. ISSN 1405–2768. Polibotánica, 34, 99–126. http://www.scielo.org.mx/scielo.php?script=sci_arttext%26pxml:id=S140527682012000200004%26lng=es%26tlng=es

[ece36113-bib-0012] Díaz‐Núñez, V. , Sosa‐Ramírez, J. , & Pérez‐Salicrup, D. R. (2016). Vegetation patch dynamics and tree diversity in a diverse conifer and oak forest in central Mexico. Botanical Science, 94(2), 229–240. 10.17129/botsci.284

[ece36113-bib-0013] Environmental Systems Research Institute . (2011). ArcGis version 10.5. Redlands, California. Available at http://www.esri.com/software/arcgis/arcgisonline

[ece36113-bib-0014] Escribano‐Ávila, G. , Calvino‐Cancela, M. , Pias, B. , Virgos, E. , Valladares, F. , & Escudero, A. (2014). Diverse guilds provide complementary dispersal services in a woodland expansion process after land abandonment. Journal of Applied Ecology, 51(6), 1701–1711. 10.1111/1365-2664.12340

[ece36113-bib-0015] Escribano‐Avila, G. , Pias, B. , Sanz‐Perez, V. , Virgos, E. , Escudero, A. , & Valladares, F. (2013). Spanish juniper gain expansion opportunities by counting on a functionally diverse dispersal assemblage community. Ecology and Evolution, 3(11), 3751–3763. 10.1002/ece3.753 24198937 PMC3810872

[ece36113-bib-0016] Escribano‐Ávila, G. , Sanz‐Perez, V. , Pias, B. , Virgos, E. , Escudero, A. , & Valladares, F. (2012). Colonization of abandoned land by *Juniperus thurifera* is mediated by the interaction of a diverse dispersal assemblage and environmental heterogeneity. PLoS ONE, 7(10), e46993 10.1371/journal.pone.0046993 23071692 PMC3468541

[ece36113-bib-0017] Fedriani, J. M. , Palomares, F. , & Delibes, M. (1999). Niche relations among three sympatric Mediterranean carnivores. Oecologia, 121, 138–148. 10.1007/s004420050915 28307883

[ece36113-bib-0018] Gelmi‐Candusso, T. A. , Bialozyt, R. , Slana, D. , Zárate Gómez, R. , Heymann, E. W. , & Heer, K. (2019). Estimating seed dispersal distance: A comparison of methods using animal movement and plant genetic data on two primate‐dispersed Neotropical plant species. Ecology and Evolution, 9(16), 8965–8977. 10.1002/ece3.5422 31462995 PMC6706201

[ece36113-bib-0019] Godínez‐Álvarez, H. , Valiente‐Banuet, A. I. , & Rojas‐Martínez, A. (2002). The role of seed dispersers in the population dynamics of the columnar cactus Neobuxbaumia tetetzo. ISSN: 0012–9658. Ecology, 2617–2629. 10.1890/00129658(2002)083[2617:TROSDI]2.0.CO;2

[ece36113-bib-0020] González‐Varo, J. P. , López‐Bao, J. V. , & Guitian, J. M. (2013). Functional diversity among seed dispersal kernels generated by carnivorous mammals. The Journal of Animal Ecology, 82(3), 562–571. 10.1111/1365-2656.12024 23228197

[ece36113-bib-0021] Graae, B. J. , Pagh, S. , & Bruun, H. H. (2004). An experimental evaluation of the Arctic fox (*Alopex lagopus*) as a seed disperser. Arctic, Antarctic, and Alpine Research, 36(4), 468–473.

[ece36113-bib-0022] Guariguata, M. R. (1999). Early response of selected tree species to liberation thinning in a young secondary forest in Northeastern Costa Rica. Forest Ecology and Management, 124(2–3), 255–261. 10.1016/s0378-1127(99)00072-9

[ece36113-bib-0023] Hämäläinen, A. , Broadley, K. , Droghini, A. , Haines, J. A. , Lamb, C. T. , Boutin, S. , & Gilbert, S. (2017). The ecological significance of secondary seed dispersal by carnivores. Ecosphere, 8(2), e01685 10.1002/ecs2.1685

[ece36113-bib-0024] Jordano, P. , Garcia, C. , Godoy, J. A. , & Garcia‐Castano, J. L. (2007). Differential contribution of frugivores to complex seed dispersal patterns. Proceedings of the National Academy of Sciences of the United States of America, 104(9), 3278–3282. 10.1073/pnas.0606793104 17360638 PMC1805555

[ece36113-bib-0025] Keeley, J. E. , Keeley, M. , & Bond, W. J. (1999). Stem demography and post‐fire recruitment of a resprouting serotinous conifer. Journal of Vegetation Science, 10, 69–76. 10.2307/3237162

[ece36113-bib-0026] Korschgen, L. J. (1969). Procedure for food‐habits analyses. The Wildlife Investigational Techniques, 233–250. http://agris.fao.org/agrissearch/search.do?recordID=US19830915895

[ece36113-bib-0027] López‐Bao, J. V. , & González‐Varo, J. P. (2011). Frugivory and spatial patterns of seed deposition by carnivorous mammals in anthropogenic landscapes: A multi‐scale approach. PLoS ONE, 6(1), e14569 10.1371/journal.pone.0014569 21297861 PMC3024974

[ece36113-bib-0028] Luna‐Ruíz, J. J. , Moreno‐Rico, O. , Sosa‐Ramírez, J. , & Sánchez‐Martínez, G. (2016). Fenología y estrategias de propagación de la manzanita en la Sierra Fría, Aguascalientes Sosa‐Ramírez J., Moreno‐Rico O., Sánchez‐Martínez G. , Luna‐Ruiz J. J., & Siqueiros‐Delgado M. E. Contribución al conocimiento ecológico del chaparral de manzanita (Arctostaphylos pungens Kunth) en la Sierra Fría, Aguascalientes. Aguascalientes: Universidad Autónoma de Aguascalientes (UAA) Retrieved from https://www.uaa.mx/direcciones/dgdv/editorial/.

[ece36113-bib-0029] Márquez‐Linares, M. A. , Jurado, E. , & González‐Elizondo, S. (2006). Algunos aspectos de la biología de la manzanita (*Arctostaphylos pungens* HBK) y su papel en el desplazamiento de bosques templados por chaparrales. ISSN 1405–9177. CIENCIA UANL, 57–64.

[ece36113-bib-0030] Martínez, A. J. , Sainos, P. , Lezama‐Delgado, E. , & Angeles‐Álvarez, G. (2007). El tamaño sí importa: Los frutos grandes de *Juniperus deppeana* Steud. (sabino) son más susceptibles a depredación por insectos. Madera Y Bosques, 13(2), 65–81.

[ece36113-bib-0031] Martínez, M. (1963). Las pináceas mexicanas. México, D. F.: Universidad Nacional Autónoma de México.

[ece36113-bib-0032] Martínez‐Vázquez, J. , María González‐Monroy, R. , & Díaz‐Díaz, D. (2010). Hábitos alimentarios del Coyote en el parque nacional Pico de Orizaba. Therya, 1, 145–154. 10.12933/therya-10-4

[ece36113-bib-0033] Matías, L. , Zamora, R. , Mendoza, I. , & Hodar, J. A. (2010). Seed dispersal patterns by large frugivorous mammals in a degraded mosaic landscape. Restoration Ecology, 18(5), 619–627. 10.1111/j.1526-100X.2008.00475.x

[ece36113-bib-0034] Monroy‐vilchis, O. , & Rubio Rodríguez, R. (2014). Guía de identificación de mamiferos terrestres del Estado de México, a través del pelo de guardia (1) (p. 115). México: Universidad Autónoma del estado de México.

[ece36113-bib-0036] Murray, K. G. , Russell, S. , Picone, C. M. , Winnettmurray, K. , Sherwood, W. , & Kuhlmann, M. L. (1994). Fruit laxatives and seed passage rates in frugivores: Consequences for plant reproductive success. Ecology, 75(4), 989–994. 10.2307/1939422

[ece36113-bib-0037] Nakashima, Y. , Inoue, E. , Inoue‐Murayama, M. , Abd Sukor, J. R. (2010). Functional uniqueness of a small carnivore as seed dispersal agents: A case study of the common palm civets in the Tabin Wildlife Reserve, Sabah, Malaysia. Oecologia, 164, 721–730. 10.1007/s00442-010-1714-1 20602116

[ece36113-bib-0038] Nogales, M. , Castañeda, I. , López‐Darias, M. , Medina, F. M. , & Bonnaud, E. (2015). The unnoticed effect of a top predator on complex mutualistic ecological interactions. Biological Invasions, 17, 1655–1665. 10.1007/s10530-014-0823-x

[ece36113-bib-0039] Nogales, M. , Medina, F. M. , & Valido, A. (1996). Indirect seed dispersal by the feral cats Felis catus in island ecosystems (Canary Islands). Ecography, 19, 3–6. 10.1111/j.1600-0587.1996.tb00149.x

[ece36113-bib-0040] Nova, J. S. (2012). The Wildlife Techniques Manual: Volume 1: Research. Volume 2: Management, 7ma edición Baltimore, MD: The Johns Hopkins University Press 686 y 414. https://books.google.com.mx/books?xml:id=PL2IHTdzSeAC%26printsec=frontcover%26redir_esc=y#v=onepage%26q%26f=false

[ece36113-bib-0041] Oliver, C. D. , & Larson, B. C. (1996). Forest stand dynamics (p. 520). New York, NY: McGraw‐Hill ISBN: 0471138339, 9780471138334. https://www.journals.uchicago.edu/doi/pdfplus/10.1086/419821

[ece36113-bib-0042] Otani, T. (2002). Seed dispersal by Japanese marten *Martes melampus* in the subalpine shrubland of northern Japan. Ecological Research, 17(1), 29–38. 10.1046/j.1440-1703.2002.00460.x

[ece36113-bib-0043] Pech‐Canche, J. , Sosa‐Escalante, J. , & Cruz, M. (2009). Guía para la identificacion de pelos de guardia de mamíferos no voladores del Estado de Yucatán, México. Revista Mexicana De Mastozoología (Nueva Época), 13(1), 7–33. 10.22201/ie.20074484e.2009.13.1.33

[ece36113-bib-0044] Perea, R. , Delibes, M. , Polko, M. , Suárez‐Esteban, A. , & Fedriani, J. M. (2013). Context‐dependent fruit–frugivore interactions: Partner identities and spatio‐temporal variations. Oikos, 122(6), 943–951. 10.1111/j.1600-0706.2012.20940.x

[ece36113-bib-0045] Rodríguez‐Estrella, R. , Moreno, A. R. , & Tam, K. G. (2000). Spring diet of the endemic ring‐tailed cat (*Bassariscus astutus* insulicola) population on an island in the Gulf of California, Mexico. Journal of Arid Environments, 44(2), 241–246. 10.1006/jare.1999.0579

[ece36113-bib-0046] Russo, S. E. , Portnoy, S. , & Augspurger, C. K. (2006). Incorporating animal behavior into seed dispersal models: Implications for seed shadows. Ecology, 87(12), 3160–3174. 10.1890/0012-9658(2006)87[3160:iabisd]2.0.co;2 17249240

[ece36113-bib-0047] Rzedowski, J. (1978). La vegetación de México. México, D.F.: Limusa http://www.academia.edu/9142430/VEGETACION_DE_MEXICO_Jerzy_Rzedowski

[ece36113-bib-0048] Sánchez‐González, R. , Martin, A. D. H. S. , Rosas‐Rosas, O. C. , & García‐Chávez, J. (2018). Diet and abundance of bobcat (*Lynx rufus*) in the Potosino‐Zacatecano Plateau, Mexico. Therya, 9(2), 10.12933/therya-18-498ISSN2007-3364

[ece36113-bib-0049] Sarasola, J. H. , Zanón‐Martínez, J. I. , Costán, A. S. , & Ripple, W. J. (2016). Hypercarnivorous apex predator could provide ecosystem services by dispersing seeds. Scientific Reports, 6, 19647 10.1038/srep19647 26791932 PMC4726145

[ece36113-bib-0050] SEDESO . (1995). Programa Integral de Manejo de la Zona Sujeta a Conservación Ecológica Sierra Fría. Aguascalientes: SEDESO.

[ece36113-bib-0051] Sosa‐Ramírez, J. , Breceda‐Solís, A. , Jiménez‐Sierra, C. L. , Iñiguez‐Dávalos, L. I. , Ortega‐Rubio, A. 2016 Capitulo XIX. Los ecosistemas de la Sierra Fría en Aguascalientes y su conservación Ortega‐Rubio, A. , Pinkus‐Rendón, M. J. , & Espitia‐ Moreno, I. C. (Editores). Las Áreas Naturales Protegidas y la Investigación Científica en México (pp. 447–471). Morelia, Michoacán: Centro de Investigaciones Biológicas del Noroeste S. C., Universidad Autónoma de Yucatán y Universidad Michoacana de San Nicolás de Hidalgo. ISBN: 978‐607‐424‐558‐5.

[ece36113-bib-0052] Suárez‐Esteban, A. , Delibes, M. , & Fedriani, J. M. (2013). Barriers or corridors? The overlooked role of unpaved roads in endozoochorous seed dispersal. Journal of Applied Ecology, 50, 767–774. 10.1111/1365-2664.12080

[ece36113-bib-0053] Villalobos Escalante, A. , Buenrostro, A. , & Vega, G. (2014). Dieta de la zorra gris *Urocyon cinereoargenteus* y su contribución a la dispersión de semillas en la costa de Oaxaca, México. Therya, 5, 355–363. 10.12933/therya-14-143

[ece36113-bib-0054] Zúñiga, A. , Muñoz‐Pedreros, A. , & Fierro, A. (2008). Dieta de *Lycalopex griseus* (GRAY, 1837) (Mammalia: Canidae) en la depresion intermedia del sur de Chile. Gayana (Concepción), 72(1), 113–116. 10.4067/S071765382008000100013

